# Associations between eating speed and food temperature and type 2 diabetes mellitus: a cross-sectional study

**DOI:** 10.3389/fnut.2023.1205780

**Published:** 2023-07-25

**Authors:** Yan Lu, Jia Liu, Johnson Boey, Ruiying Hao, Guopeng Cheng, Wentan Hou, Xinhui Wu, Xuan Liu, Junming Han, Yuan Yuan, Li Feng, Qiu Li

**Affiliations:** ^1^Department of Endocrinology, Shandong Provincial Hospital, Shandong University, Jinan, Shandong, China; ^2^Department of Endocrinology, Shandong Provincial Hospital Affiliated to Shandong First Medical University, Jinan, Shandong, China; ^3^Department of Podiatry, National University Hospital Singapore, Singapore, Singapore; ^4^Department of Biostatistics, School of Public Health, Shandong University, Jinan, Shandong, China; ^5^Department of Clinical Nutrition, Shandong Provincial Hospital Affiliated to Shandong First Medical University, Jinan, Shandong, China

**Keywords:** type 2 diabetes mellitus, eating behaviors, risk factors, Chinese adults, cross-sectional study

## Abstract

**Objective:**

This study aimed to evaluate the relationship between eating speed and food temperature and type 2 diabetes mellitus (T2DM) in the Chinese population.

**Methods:**

A cross-sectional survey was conducted between December 2020 to March 2022 from the department of Endocrinology at the Shandong Provincial Hospital. All recruited participants were asked to complete structured questionnaires on their eating behaviors at the time of recruitment. Clinical demographic data such as gender, age, height, weight, familial history of T2DM, prevalence of T2DM and various eating behaviors were collected. Univariate and multivariate logistic regression analyses were used to analyze the associations between eating behaviors and T2DM.

**Results:**

A total of 1,040 Chinese adults were included in the study, including 344 people with T2DM and 696 people without T2DM. Multivariate logistic regression analysis of the general population showed that gender (OR = 2.255, 95% CI: 1.559–3.260, *p* < 0.001), age (OR = 1.091, 95% CI: 1.075–1.107, *p* < 0.001), BMI (OR = 1.238, 95% CI: 1.034–1.483, *p* = 0.020), familial history of T2DM (OR = 5.709, 95% CI: 3.963–8.224, *p* < 0.001), consumption of hot food (OR = 4.132, 95% CI: 2.899–5.888, *p* < 0.001), consumption of snacks (OR = 1.745, 95% CI: 1.222–2.492, *p* = 0.002), and eating speed (OR = 1.292, 95% CI:1.048–1.591, *p* = 0.016) were risk factors for T2DM.

**Conclusion:**

In addition to traditional risk factors such as gender, age, BMI, familial history of T2DM, eating behaviors associated with Chinese culture, including consumption of hot food, consumption of snacks, and fast eating have shown to be probable risk factors for T2DM.

## 1. Introduction

The prevalence of type 2 diabetes mellitus (T2DM) in China has increased nearly five-fold over the past three decades, from 2.5% in 1994 to 12.4% in 2018 (1, 2). Emerging evidence has suggested dietary and lifestyle changes driven by rapid urbanization may explain the surge in prevalence of T2DM in China (3). The rich variety of food prompts people to pay more attention to the color, aroma and taste of food, while ignoring the rationality of the dietary structure, which directly affects the body’s calorie intake and leads to a significant increase in the incidence of type 2 diabetes and other related metabolic diseases. Previous studies have mainly focused on the correlation of calorie intake and dietary patterns with incidence of T2DM (4–8). Recent ADA guideline recommend combination of behavioral, dietary and lifestyle changes to maintain daily calories expenditure of between 500 and 750 Kcal (9). It was noted that scanty researches have evaluated the impact of eating behaviors on T2DM (10).

In spite of the cultural diversity within China, rice and wheaten food have been considered as the staple food (11). The belief in the consumption of hot food will provide more energy to the body especially during the winters and bring about better taste, has become the embodiment of traditional Chinese food culture (12). With rapid urbanization of Chinese society, the social behavior pattern of people’s food consumption has undergone a paradigm shift, gradually shifting from the steaming and boiling of food to the frying of food (13). Hot food restaurants represented by Chinese hot pot, barbecue, and hot soup noodles and various snack stores have developed rapidly throughout the country, (14) prompting people to eat hot food and snacks more frequently, and people’s eating speed is gradually accelerating. Hot-pot restaurants are becoming increasingly popular among university students in many Chinese cities, especially in Chongqing, and they are also popular among Chinese adults aged 18–59 (15). Regarding the relationship between hot food and T2DM, previous studies have found that cooking foods at high temperatures can induce the formation of advanced glycation end products (AGEs), high levels of AGEs can promote insulin resistance and increase the incidence of T2DM, and modulating cooking methods can reduce AGEs levels, improve insulin resistance and delay the development of diabetes (16, 17). In addition, a previous study found that a diet that was based on high-heat-treated foods could increase markers associated with an enhanced risk of T2DM and cardiovascular diseases in healthy people (18). This study also found that replacing high-heat-treatment techniques by mild cooking techniques may help to positively modulate biomarkers associated with an increased risk of diabetes mellitus and cardiovascular diseases (18). Based on the above background, we conducted this cross-sectional study to examine the associations of selected eating behaviors and T2DM in the Chinese population. The results from this study will enable the health ministry to evaluate current preventive strategies for the management T2DM.

## 2. Materials and methods

### 2.1. Study participants

A population-based cross-sectional study among Chinese adults was conducted from December 2020 to March 2022 using convenience sampling. In this study, all recruited study participants were aged ≥20 years old and the diagnostic criteria for T2DM met the 2022 American Diabetes Association (ADA) diagnostic criteria for T2DM (9). At the same time, these participants need to exhibit the ability to complete the questionnaire by themselves using a smartphone and they all volunteered to participate in the survey and signed relevant informed consent forms. The exclusion criteria for study participants were gestational diabetes mellitus, type 1 diabetes mellitus and other special types of diabetes mellitus, severe hepatic and renal insufficiency, hyperthyroidism, Cushing’s syndrome, and other endocrine diseases and mental disorders.

### 2.2. Questionnaire and definitions

The questionnaire used in this study was a structured questionnaire designed by the researchers around the hypothesis of the role of eating behaviors on T2DM based on the unique eating behavior characteristics of Chinese adults (19, 20). In addition to collecting the demographics of study participants, such as gender, age, height, weight, familial history of T2DM, the questionnaire evaluated various eating behaviors (including consumption of hot food, consumption of snacks and eating speed) and the prevalence of T2DM.

In accordance to the Guidelines for Prevention and Control of Overweight and Obesity in Chinese Adults, Chen and Lu (21) the body mass index (BMI) of study participants were divided into the underweight group (BMI < 18.5 kg/m^2^), the normal weight group (18.5 kg/m^2^ ≤ BMI < 24 kg/m^2^), the overweight group (24 kg/m^2^ ≤ BMI < 28 kg/m^2^) and the obese group (BMI ≥ 28.0 kg/m^2^). In addition, the eating speed was estimated by the meal duration of average per meal of participants, and was divided into the superfast group (meal duration<10 min), the fast group (10 min ≤ meal duration<20 min), the medium group (20 min ≤ meal duration<30 min), and the slow group (meal duration≥30 min) (22–24).

### 2.3. Data collection

This study distributed and collected structured questionnaires about Chinese adults’ eating behaviors and T2DM online. All recruited study participants were briefed on the purpose of the study and informed consent were obtained. To eliminate duplicate data, we limited one IP address per submission and each study participant was required to log in the online survey platform with a personal mobile phone number that was a real-name system. The completed questionnaires were automatically stored as secured data files, where two researchers were given access for further verification of these data to ensure the reliability of the survey data. In this study, 1,140 questionnaires were collected. After excluding invalid questionnaires with missing items or duplicate answers, a total of 1,040 valid questionnaires were returned, and the effective response rate was 91%.

### 2.4. Statistical analysis

Statistical analysis was performed using SPSS 25.0 statistical software. The measurement data of normal distribution was expressed as mean ± standard deviation, and the comparison between two groups was performed by independent sample t test. The measurement data of non-normal distribution was expressed by the median (interquartile interval), and the comparison between two groups was conducted by nonparametric test. The chi-square (*χ*^2^) test was used to compare the enumeration data between two groups. The eating behavior variable with statistically significant difference (*p* < 0.05) in the difference analysis was selected as the independent variable, other factors that may affect the prevalence of T2DM were taken as covariates, and whether a study participant had T2DM was taken as the dependent variable. Univariate logistic regression analysis and multivariate logistic regression analysis were used to analyze the association between different eating behaviors and T2DM in Chinese adults, and the odds ratio (OR) and 95% confidence interval (95% CI) were calculated. *p* < 0.05 was considered as statistically significant.

## 3. Results

### 3.1. Baseline data for study participants

A total of 1,040 Chinese adults were enrolled into the study, of which 344 people were diagnosed with T2DM and remaining 696 people were non-T2DM. The results of the difference analysis showed that age (*p* < 0.05), gender (*p* < 0.05), body mass index (BMI) (*p* < 0.05), familial history of T2DM (*p* < 0.05), consumption of hot food (*p* < 0.05), consumption of snacks (*p* < 0.05), and eating speed (*p* < 0.05) were significantly different between the T2DM group and the non-T2DM (Table 1). The mean age of the T2DM group was 48.14 ± 14.45 years old, which was significantly higher than the average age of the non-T2DM group (Table 1). In people with T2DM, the proportion of males accounted for 64.24%, which was significantly higher than females. And the overweight people (24 kg/m^2^ ≤ BMI < 28 kg/m^2^) accounted for 37.2%, which was the highest proportion in people with T2DM (Table 1). In addition, the fast group (10 min ≤ meal duration<20 min) accounted for 48.8%, which was the highest proportion in people with T2DM (Table 1).

**Table 1:** General information of Non-T2DM group and T2DM group.

Variables		Non-T2DM	T2DM	p*
		(*n* = 696)	(*n* = 344)	
Age, year		35.79 ± 10.34	48.14 ± 14.45	<0.001
Gender (%)	Male	49.7	64.2	
	Female	50.2	35.8	<0.001
BMI, kg/m^2^, (%)	<18.5	22.4	14.8	
	18.5–24	43.1	26.8	<0.001
	24–28	22.6	37.2	
	≥28	11.9	21.2	
Familial history of T2DM (%)	No	85.8	49.7	<0.001
	Yes	14.2	50.3	
Consumption of hot food? (%)	No	79.0	45.1	<0.001
	Yes	21.0	54.9	
Consumption of snacks? (%)	No	58.1	46.2	<0.001
	Yes	41.9	53.8	
Eating speed in minute (%)	<10	14.5	24.1	
	10–20	46.0	48.8	<0.001
	20–30	30.9	19.5	
	≥30	8.6	7.6	

*Independent sample t test was used to compare the measurement data of normal distribution between two groups, and *χ*^2^ test was used to compare the enumeration data between two groups.

### 3.2. Univariate logistic regression analysis of eating behaviors and T2DM in Chinese adults

Taking the variables with statistically significant differences (*p* < 0.05) in Table 1 as the independent variables and whether a participant developed T2DM (Yes = 1, No = 0) as the dependent variable, a univariate logistic regression analysis was carried out, in which *P* represents the probability of developing T2DM and 1-*P* represents the probability of not developing T2DM. The results showed that gender (OR = 1.818, 95% CI: 1.393–2.371, *p* < 0.001), age (OR = 1.081, 95% CI: 1.068–1.094, *p* < 0.001), BMI (OR = 1.561, 95% CI: 1.360–1.793, *p* < 0.001), familial history of T2DM (OR = 6.101, 95% CI: 4.520–8.234, *p* < 0.001), consumption of hot food (OR = 4.593, 95% CI: 3.472–6.078, *p* < 0.001), consumption of snacks (OR = 1.610, 95% CI:1.241–2.088, *p* < 0.001), and eating speed (OR = 1.398, 95% CI: 1.193–1.639, *p* < 0.001) were risk factors for T2DM in Chinese adults (Table 2).

**Table 2:** Univariate logistic regression analysis of eating behaviors and T2DM in Chinese adults.

Independent variables	OR (95% CI)	*p*
Gender	1.818 (1.393–2.371)	<0.001
Age, year	1.081 (1.068–1.094)	<0.001
BMI, kg/m^2^	1.561 (1.360–1.793)	<0.001
Familial history of T2DM	6.101 (4.520–8.234)	<0.001
Consumption of hot food	4.593 (3.472–6.078)	<0.001
Consumption of snacks	1.610 (1.241–2.088)	<0.001
Eating speed, minute	1.398 (1.193–1.639)	<0.001

### 3.3. Multivariate logistic regression analysis of eating behaviors and T2DM in Chinese adults

As mentioned above, this study further took the variables with statistically significant differences (*p* < 0.05) in univariate logistic regression analysis as the independent variables and whether a participant developed T2DM (Yes = 1, No = 0) as the dependent variable. A multivariate logistic regression analysis was carried out, in which *P* represents the probability of developing T2DM and 1-*P* represents the probability of not developing T2DM. The results showed that gender (OR = 2.255, 95% CI: 1.559–3.260, *p* < 0.001), age (OR = 1.091, 95% CI: 1.075–1.107, *p* < 0.001), BMI (OR = 1.238, 95% CI: 1.034–1.483, *p* = 0.020), familial history of T2DM (OR = 5.709, 95% CI: 3.963–8.224, *p* < 0.001), consumption of hot food (OR = 4.132, 95% CI: 2.899–5.888, *p* < 0.001), consumption of snacks (OR = 1.745, 95% CI: 1.222–2.492, *p* = 0.002), and eating speed (OR = 1.292, 95% CI:1.048–1.591, *p* = 0.016) were risk factors for T2DM (Table 3).

**Table 3:** Multivariate logistic regression analysis of eating behaviors and type 2 diabetes in Chinese adults.

Independent variables	OR (95% CI)	*p*
Gender	2.255 (1.559–3.260)	<0.001
Age, year	1.091 (1.075–1.107)	<0.001
BMI, kg/m^2^	1.238 (1.034–1.483)	0.020
Familial history of T2DM	5.709 (3.963–8.224)	<0.001
Consumption of hot food	4.132 (2.899–5.888)	<0.001
Consumption of snacks	1.745 (1.222–2.492)	0.002
Eating speed, minute	1.292 (1.048–1.591)	0.016

## 4. Discussion

T2DM is a metabolic disorder characterized by chronic hyperglycemia with multiple systemic complications involving the eyes, heart, kidney and the feet. Though current studies indicate that an appropriate dietary structure is imperative for prevention of T2DM, Inter Act Consortium (25) and Toi et al. (26) dietary intake cannot fully explain the increasing incidence of T2DM in China. As China embarks on urbanization riding on the waves of globalization, the social lifestyle and eating behaviors of people continues to evolve. This study explored the associations between various eating behaviors such as consumption of hot food, consumption of snacks, and eating speed with T2DM in Chinese adults. Previous epidemiological studies have established that advancing age, Sun et al. (27), Li et al. (28), Zhang et al. (29), Chen et al., (30), King et al. (31), DECODE Study Group (32), BMI (33–35), and genetic factors (36–39) may predispose one to develop T2DM. This study also showed that age, BMI and familial history of T2DM were risk factors for T2DM, which were consistent with those of previous studies mentioned earlier. In this study, the mean age of T2DM group was 48.14 ± 14.45 years old and was significantly higher than the average age of the non-T2DM group. In addition, in the T2DM group, the overweight people accounted for 37.2%, which accounted for the highest proportion. This may be related to the excessive accumulation of fat in the body due to weight gain, resulting in a disorder in the secretion of adipokines. Previous studies have found that in overweight and obese patients, the levels of leptin secreted by adipocytes significantly increased (40, 41). Under the long-term stimulation of high concentrations of leptin, the receptor responsiveness of the islets β cells of the body decreased, thereby reducing the inhibitory effect of leptin on insulin synthesis and increasing insulin secretion, leading to hyperinsulinemia and insulin resistance (40, 41). Zulfania et al. (42) found that leptin levels in T2DM patients were positively correlated with HbA1c and BMI, and elevated serum leptin levels may be a risk factor for T2DM. In addition, resistin secreted by fat cells can inhibit insulin signaling, leading to insulin resistance and the development of T2DM (43).

In recent years, with the rapid development of the social economy, various hot food restaurants represented by Chinese hot pot, barbecue, and hot soup noodles are rapidly emerging in China (12, 14). Affected by the surrounding dietary environment, people’s frequency of eating hot food and snacks has gradually increased, and their eating speed has also gradually increased (19, 20). A number of studies have found that poor chewing and eating fast are related to obesity, type 2 diabetes mellitus and metabolic syndrome in children and adults (44–47). Research has found that chewing frequency can regulate the body’s energy intake and blood sugar levels by affecting the secretion of gastrointestinal hormones. The concentration of plasma glucagon like peptide-1 (GLP-1) when people chewed 40 times per meal was significantly higher than that when people chewed 15 times per meal, while the concentration of ghrelin when people chewed 40 times per meal was significantly lower than that when people chewed 15 times per meal (48). In gastrointestinal hormones, ghrelin can promote the body’s appetite and increase blood sugar, while GLP-1 can inhibit the body’s appetite and lower blood sugar (48). Therefore, properly increasing the chewing frequency can increase the concentration of GLP-1 and reduce the concentration of ghrelin in the body, which is conducive to reducing the energy intake of the body and lowering the blood sugar level. In this study, there was a significant difference in eating speed between the T2DM group and the non-T2DM group (*p* < 0.05). The fast group (10 min ≤ meal duration<20 min) accounted for the highest proportion in people with T2DM, while the slow group (meal duration≥30 min) accounted for the lowest proportion in people with T2DM. The multivariate logistic regression analysis showed that eating speed was a risk factor for T2DM. This suggests that people should increase their chewing frequency and slow down their eating speed during daily meals to reduce the prevalence of T2DM.

Regarding the relationship between hot food and T2DM, previous studies have found that cooking foods at high temperatures can induce the formation of advanced glycation end products (AGEs), high levels of AGEs can promote insulin resistance and increase the incidence of T2DM, and modulating cooking methods can reduce AGEs levels, improve insulin resistance and delay the development of diabetes (16, 17). AGEs also can enhance the response and appetite of postprandial ghrelin (49) and significantly reduce postprandial leptin levels in patients with T2DM (50). Among them, leptin can act on leptin receptors in the arcuate nucleus of the hypothalamus and inhibits neuropeptide Y/agouti-related protein (NPY/AgRP) neurons and activate proopiomelanocortin/cocaine-and amphetamine-regulated transcript (POMC/CART) neurons, which suppress the body’s appetite, thereby reducing the body’s food intake and lowering blood sugar levels (51). Therefore, consumption of hot food can affect the body’s appetite by affecting the secretion of gastrointestinal hormones, thereby affecting the body’s blood sugar levels. In this study, there was a significant difference in consumption of hot food between the T2DM group and the non-T2DM group (*p* < 0.05), and the multivariate logistic regression analysis of the general population showed that consumption of hot food was a risk factor for T2DM. Therefore, in daily dietary management, people should reduce the temperature of the food they ingest, reduce the frequency of eating hot food, so as to reduce the prevalence of T2DM.

In addition, with the rise of various snack convenience stores, people often eat various snacks without being hungry after meals. In fact, consumption of snacks is a kind of hedonic eating that is not regulated by metabolic feedback and is related to cognitive, reward and emotional factors (52). The reward effect of food will promote the body to continue eating, increase the body’s total energy intake, and affect changes in weight and blood sugar. Bauer et al. (53) conducted an FFQ (Food Frequency Questionnaire) to collect dietary data on 20,835 overweight and obese Dutch individuals, and the results showed that a model dominated by soft drinks, chips and snacks was associated with a higher risk of T2DM. The multivariate logistic regression analysis of this study also showed that consumption of snacks was a risk factor for T2DM. Therefore, in the daily diet management, people should try to avoid being affected by the surrounding dietary environment, reduce the frequency of going to snack convenience stores and eating snacks, so as to reduce the prevalence of T2DM.

## 5. Strengths and limitations

This study systematically explored the relationship between consumption of hot food, consumption of snacks and eating speed and T2DM for the first time, and confirmed that gender, age, BMI, familial history of T2DM, consumption of hot food, consumption of snacks and eating fast were risk factors for T2DM. In clinical practice, it is difficult for patients to assess the proportion of specific nutrients (carbohydrate, lipid and protein) in each food, and it is difficult to eat strictly according to specific dietary structure, which increases the difficulty of dietary management for T2DM. Taking the results of this study as a guide, we can more directly observe and intervene with people’s eating behaviors, such as encouraging people to slow down the speed of eating, reduce the temperature of food and avoid eating snacks to reduce the risk of T2DM, which provides new ideas for the dietary management of T2DM. However, this study is a cross-sectional study. Its findings can only provide the risk factors of T2DM, and cannot be used to infer the causal relationship of the disease. It cannot well confirm the causal relationship between the eating behaviors of Chinese adults and T2DM. Prospective cohort study or intervention test should be conducted in the future to further verify this research conclusion. In addition, the types of eating behaviors discussed in this study are relatively few. In the future, we can further explore the relationship between more eating behaviors and T2DM, and provide a more comprehensive dietary management strategy for the prevention and treatment of T2DM.

## 6. Conclusion

This study confirmed that gender, age, BMI, familial history of T2DM, consumption of hot food, consumption of snacks, and eating speed were factors for T2DM in Chinese adults. On the one hand, the results of this study suggest that we should reflect on and control some unhealthy eating behaviors in the traditional eating behaviors from the social level to curb the rising trend of T2DM in China. On the other hand, in clinical practice, we can directly observe and intervene with people’s eating behaviors, such as encouraging people to increase chewing frequency, slow down eating speed, reduce the temperature of food and avoid eating snacks to reduce the risk of T2DM.

## Data availability statement

The original contributions presented in the study are included in the article/Supplementary material, further inquiries can be directed to the corresponding author.

## Ethics statement

The studies involving human participants were reviewed and approved by Shandong Provincial Hospital’s Ethics Committee for Biomedical Research Involving Human Beings. The patients/participants provided their written informed consent to participate in this study. Written informed consent was obtained from the individual(s) for the publication of any potentially identifiable images or data included in this article.

## Author contributions

YL: conceived and designed the study, collected and managed the data, analyzed and interpreted the data, drafted the initial manuscript, and edited the final manuscript. JL: co-analyzed the data and contributed to discussions about the results, and critically revised and edited the final manuscript. JB: revised and edited the final manuscript. RH: co-managed the data, contributed to discussions about the results, and edited the final manuscript. GC: screened and recruited participants and contributed to discussions about the results. WH, XW, XL, and YY: contributed to discussions about the results. JH: provided statistical expertise in the analysis and interpretation of the data, contributed to discussions about the results. LF: contributed to the conception of the study and critically revised the final manuscript. QL: co-designed the study, contributed to discussions about the results, critically reviewed and edited the final manuscript and was the guarantor of this work and took responsibility for the integrity of the data and the accuracy of the data analysis. All authors read and approved the final manuscript.

## Funding

The present study was supported by grants from the National Natural Science Foundation of China (82070861 to QL), the Natural Science Foundation of Shandong Province (ZR2019MH061 to QL), and the National Natural Science Foundation of China (81770785 to JL).

## Conflict of interest

The authors declare that the study was conducted in the absence of any commercial or financial relationships that could be construed as a potential conflict of interest.

## Publisher’s note

All claims expressed in this article are solely those of the authors and do not necessarily represent those of their affiliated organizations, or those of the publisher, the editors and the reviewers. Any product that may be evaluated in this article, or claim that may be made by its manufacturer, is not guaranteed or endorsed by the publisher.
